# Astragalus Complanatus Ethanol Attenuates Septic Shock by Exerting Anti-Inflammatory Effects on Macrophages

**DOI:** 10.3390/ijms25010384

**Published:** 2023-12-27

**Authors:** Yo Sep Hwang, Jeewon Lim, Hyang Ran Yoon, Seong-Hoon Park, Aeyung Kim, Jun-Pil Jang, Hee Jun Cho, Hee Gu Lee

**Affiliations:** 1 Immunotherapy Research Center, Korea Research Institute of Bioscience and Biotechnology, Yuseong-gu, Daejeon 34141, Republic of Korea; hys8520@kribb.re.kr (Y.S.H.); ljw8796@kribb.re.kr (J.L.); yhr1205@kribb.re.kr (H.R.Y.); 2 Department of Biomolecular Science, KRIBB School of Bioscience, Korea University of Science and Technology (UST), Yuseong-gu, Daejeon 34113, Republic of Korea; 3 Genetic and Epigenetic Toxicology Research Group, Korea Institute of Toxicology, Daejeon 34114, Republic of Korea; seonghoon.park@kitox.re.kr; 4 Korean Medicine Application Center, Korea Institute of Oriental Medicine, Daegu 41062, Republic of Korea; aykim71@kiom.re.kr; 5 Chemical Biology Research Center, Korea Research Institute of Bioscience and Biotechnology, Cheongju 28116, Republic of Korea; jpjang@kribb.re.kr

**Keywords:** *Astragalus complanatus*, sepsis, inflammation, macrophages, NF-κB

## Abstract

Sepsis is a systemic inflammatory syndrome that results in multiple-organ failure caused by a dysregulated host immune response to microbial infection. Astragali complanati semen extract (ACSE) exhibits pharmacological activities, including antioxidant, anticancer, antiaging, and anti-diabetes effects. It is widely used in traditional medicine to treat liver and kidney diseases; however, the protective effect of ACSE on sepsis and its mechanisms are unknown. In the present study, we investigated the anti-inflammatory effects and potential mechanisms of the action of ACSE on sepsis. We show that ACSE improved survival rates in mouse models of acute sepsis induced by CLP (cecal ligation and puncture) and LPS stimulation. ACSE administration decreased aspartate aminotransferase (AST) and alanine aminotransferase (ALT) in sepsis-induced mice. Furthermore, ACSE reduced the levels of nitric oxide (NO), tumor necrosis factor-α (TNF-α), interleukin-1β (IL-1β), and interleukin-6 (IL-6) in the serum of septic mice. ACSE treatment inhibited the expression of these proinflammatory genes in LPS-stimulated J774 macrophages. Moreover, ACSE inhibited the phosphorylation of the IκB kinase (IKK) and the nuclear translocation of p65 NF-κB by LPS stimulation in macrophages. These results reveal the mechanism underlying the protective effect of ACSE against sepsis by inhibiting NF-κB activation and suggest that ACSE could be a potential therapeutic candidate to treat acute inflammatory diseases.

## 1. Introduction

Sepsis is a systemic inflammatory disease caused by a dysregulated host response to bacterial infection [1]. Sepsis remains a major cause of health loss worldwide, with ap-proximately 48.9 million patients with sepsis and a mortality rate of 19.7% [2]. Sepsis is difficult to treat due to problems with early identification and diagnosis, sepsis heterogeneity, antibiotic resistance, multiple organ dysfunction syndrome (MODS), and immune system dysregulation. For this reason, despite advances in medicine, sepsis remains an unresolved public health problem worldwide. MODS is a major cause of mortality in patients and the excessive production of inflammatory factors and cytokines can lead to the development of acute lung injury and acute respiratory distress syndrome [3,4,5]. Therefore, anti-inflammatory agents that control excessive immune responses may be the key to reducing sepsis mortality.

Macrophages play an important role in various pathophysiological processes in sepsis [6]. During early sepsis, macrophages can detect pathogen-associated molecular patterns (PAMPs) and damage-associated molecular patterns (DAMPs) through pattern-recognition receptors (PRRs), which initiate a series of immune cell activations, resulting in a severe and sustained inflammatory response [3]. Lipopolysaccharides (LPS) are important cell wall constituents of gram-negative bacteria and are strong inducers of inflammatory responses [7]. Macrophages recognize LPS via toll-like receptor 4 (TLR4), a well-characterized PRR [8]. After recognizing LPS, TLR4 activates nuclear factor-κB (NF-κB), mitogen-activated protein kinase (MAPK), and signal transducer and activator of transcription (STAT) signaling pathways [9,10]. These signaling pathways promote the production of proinflammatory cytokines and mediators, such as tumor necrosis factor-α (TNF-α), interleukin-1β (IL-1β), interleukin-6 (IL-6), and nitric oxide (NO), which facilitates the clearance of pathogenic microorganisms [11,12]. Although the regulated activation of macrophages plays an essential role in host defenses against bacterial infections, excessive activation of macrophages causes several inflammation-related diseases, such as asthma and endotoxin-induced multiple-organ injury [13,14]. Therefore, it is important to control excessive inflammatory responses using appropriate drugs and treatments.

Astragali Complanati Semen (ACS) is a seed of *Astragalus complanatus*. It has been commonly used as an herbal medicine to treat several diseases, such as muscle, renal, liver, and reproductive system diseases in Korea and China [15]. ACS extracts (ACSE) contain fatty acids, amino acids, polysaccharides, flavonoids, and trace elements, and have pharmacological activities, including antioxidant, anticancer, and antiaging effects [16,17]. Therefore, ACSE have been reported to exert multiple therapeutic effects on various chronic diseases, such as cardiovascular diseases, diabetes, and cancers [18,19]. However, the effects of ACSE on sepsis have not been experimentally determined. In this study, we investigated the antiseptic effects of ACSE using LPS- and CLP-induced sepsis mouse models and explored their potential mechanisms in LPS-stimulated macrophages.

## 2. Results

### 2.1. ACSE Increases Survival Outcomes and Alleviates Liver Injury in Sepsis Mouse Models

**Figure 1:**
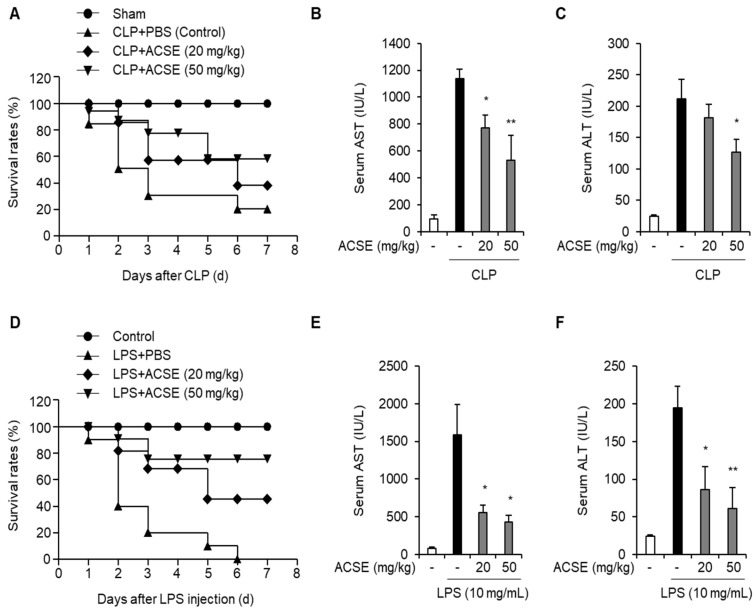
ACSE increases survival outcomes and attenuates live injury in CLP- and LPS-induced septic mice. C57BL/6 mice were orally administered with ACSE (20 or 50 mg/kg) or PBS as a vehicle control daily for seven days before CLP operation or LPS administration (n = 10 per group) and normal mice were used as a negative control. Serum samples were collected 12 h after CLP surgery or LPS administration. (**A**) Survival curve analysis of CLP mice. (**B**,**C**) The serum levels of AST and ALT of CLP mice. (**D**) Survival curve analysis of LPS-induced septic mice. (**E**,**F**) The serum levels of AST and ALT of LPS-induced septic mice. * *p* < 0.05 and ** *p* < 0.01.

The CLP-induced sepsis model is one of the most reliable animal disease models used to mimic human sepsis [20,21]. Therefore, we established a CLP mouse model using C57BL/6 mice to determine the effects of ACSE on sepsis. After oral administration of ACSE (20 or 50 mg/kg) for 7 days, the mice underwent CLP. Survival rates were lower in the CLP-only group than in the sham group. However, administration of ACSE increased the survival rates of mice with CLP compared to those in the CLP-only mice (Figure 1A). Furthermore, we investigated the protective effect of ACSE by measuring serum levels of AST and ALT as liver injury factors. AST and ALT levels were elevated in CLP-only mice, but were significantly reduced in ACSE-treated CLP mice (Figure 1B,C).

We explored the anti-septic effects of ACSE against LPS-induced septic shock in mice. Mice were injected with LPS after oral administration of ACSE for 7 days, and the mortality of the mice was measured. The survival rates of the ACSE-treated groups improved substantially compared to those of mice treated with LPS (Figure 1D). Furthermore, serum levels of AST and ALT increased in mice injected with LPS, whereas ACSE administration significantly reduced these enhancements (Figure 1E,F). These results suggest that ACSE prolongs survival rates and alleviates liver injury in mice with CLP- and LPS-induced sepsis.

### 2.2. ACSE Attenuates Inflammatory Responses in Septic Mice

**Figure 2:**
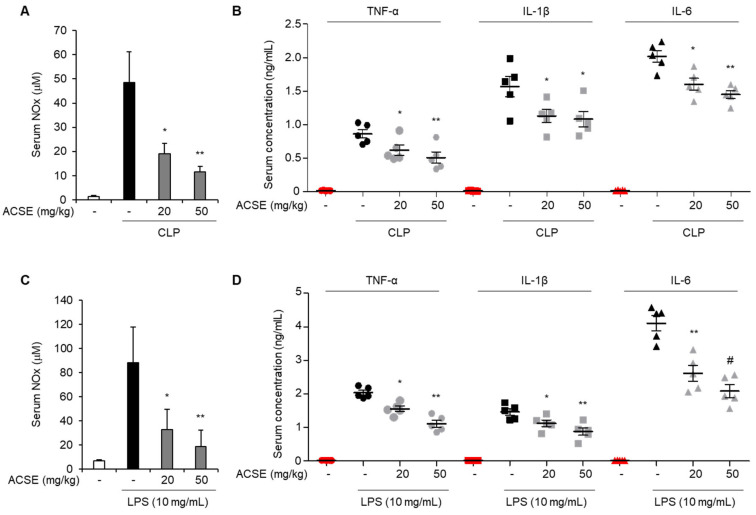
ACSE suppresses inflammatory mediators production in the serum of septic mice. C57BL/6 mice were orally administered with ACSE (20 or 50 mg/kg) or PBS as a vehicle control daily for seven days before CLP operation or LPS administration (n = 10 per group) and normal mice were used as a negative control. Serum samples were collected 12 h after CLP surgery or LPS administration. (**A**) The serum level of NO of CLP mice. (**B**) The serum levels of TNF-α, IL-1β, and IL-6 in CLP mice. (**C**) The serum level of NO of LPS-induced septic mice. (**D**) Serum levels of TNF-α, IL-1β, and IL-6 in LPS-induced septic mice. * *p* < 0.05, ** *p* < 0.01, and ^#^ *p* < 0.001.

Excessive inflammatory responses are a major clinical feature of sepsis [5]. We investigated the effect of ACSE by determining NO, TNF-α, IL-1β, and IL-6 levels in the serum of CLP-induced septic mice. As shown in Figure 2A, serum NO levels were markedly increased in CLP mice compared to those of the sham group. However, ACSE administration markedly reduced CLP-induced NO serum levels. Furthermore, TNF-α, IL-1β, and IL-6 levels in serum were also increased in CLP mice compared to those of the sham group, but these increases were reduced by ACSE administration (Figure 2B). Next, we evaluated the effects of ACSE on LPS-induced sepsis in mice. Consistently with the result observed in CLP mice, LPS treatment increased the serum levels of NO, TNF-α, IL-1β, and IL-6. However, ACSE administration markedly decreased the levels of these mediators, which were increased by LPS treatment (Figure 2C,D).

### 2.3. ACSE Suppresses the Expression of Inflammatory Mediators Induced by LPS in Macrophages

**Figure 3:**
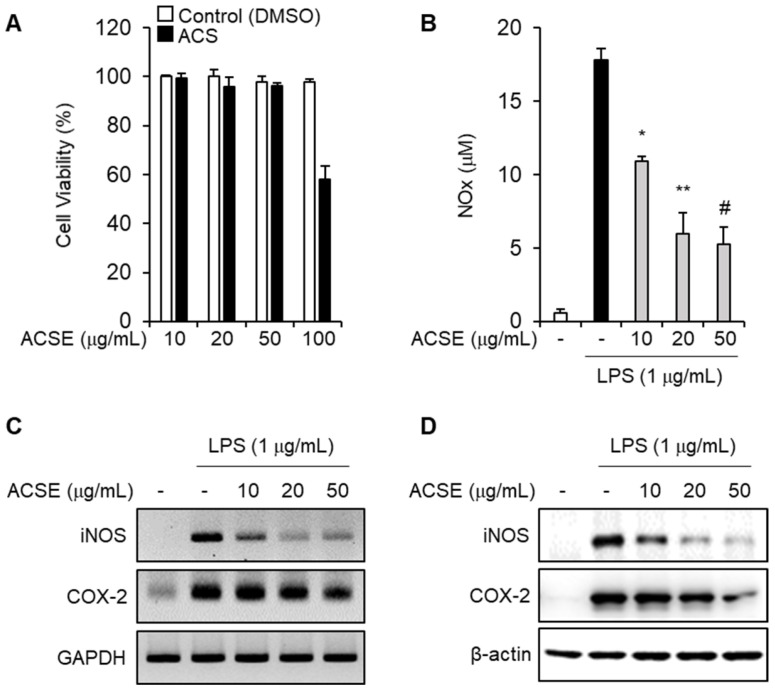
ACSE attenuates NO production in LPS-stimulated macrophages. (**A**) J774 cells were treated with indicated concentrations of ACSE for 24 h. The effects of ACSE on cell viability were measured via WST-1 assay. (**B**–**D**) J774 cells were pretreated with indicated concentrations of ACSE and then stimulated with 1 µg/mL of LPS for 24 h. (**B**) The effect of ACSE on NO production. (**C**,**D**) The effects of ACSE on the expression of iNOS and COX-2 mRNAs (**C**) and proteins (**D**). * *p* < 0.05, ** *p* < 0.01, and ^#^ *p* < 0.001.

Macrophages secrete several inflammatory mediators and induce immune responses in sepsis [11]. First, we determined the optimal concentration of ACSE to investigate whether ACSE modulates LPS-induced inflammatory responses in macrophages. Treatment with 10, 20, or 50 µg/mL ACSE did not affect the viability of J774 macrophages (Figure 3A). J774 cells were pretreated with ACSE and then stimulated with 1 µg/mL of LPS to measure the NO levels in the culture medium. ACSE pretreatment significantly inhibited LPS-induced NO production in a concentration-dependent manner (Figure 3B). Furthermore, LPS treatment induced the expression of iNOS and COX-2 mRNA and proteins, which was also suppressed by ACSE (Figure 3C,D).

**Figure 4:**
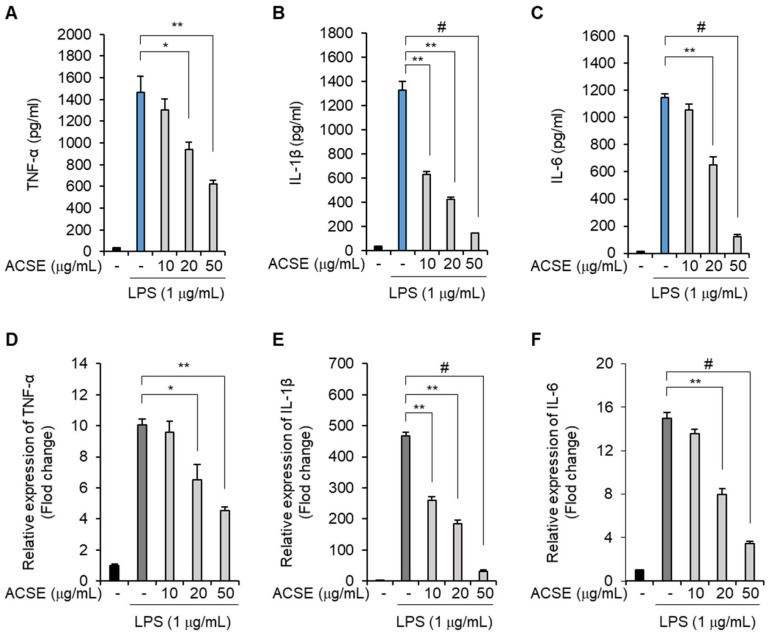
ACSE suppresses the expression of inflammatory cytokines in LPS-stimulated macrophages. J774 cells were pretreated with indicated concentrations of ACSE and then stimulated with 1 µg/mL of LPS for 24 h. (**A**–**C**) The effects of ACSE on TNF-α, IL-1β, and IL-6 production levels in the culture media of LPS-stimulated J774 cells. (**D**–**F**) The effects of ACSE on TNF-α, IL-1β, and IL-6 expression in LPS-stimulated J774 cells. * *p* < 0.05, ** *p* < 0.01, and ^#^ *p* < 0.001.

J774 macrophages were pretreated with ACSE and stimulated with LPS to evaluate its effects on the LPS-mediated production of inflammatory cytokines. LPS stimulation increased the TNF-α, IL-1β, and IL-6 levels in culture media. However, ACSE suppressed LPS-induced TNF-α production (Figure 4A). Furthermore, ACSE pretreatment considerably suppressed the LPS-induced production of IL-1β and IL-6 (Figure 4B,C). The transcriptional regulation of these cytokines was then analyzed using real-time RT-PCR. ACSE pretreatment significantly decreased the mRNA expression of TNF-α, IL-1β, and IL-6 (Figure 4D–F), suggesting that ACSE transcriptionally inhibits the LPS-induced expression of proinflammatory cytokines in macrophage cells.

### 2.4. ACSE Attenuates NF-κB Activation by LPS Stimulation in J774 Cells

**Figure 5:**
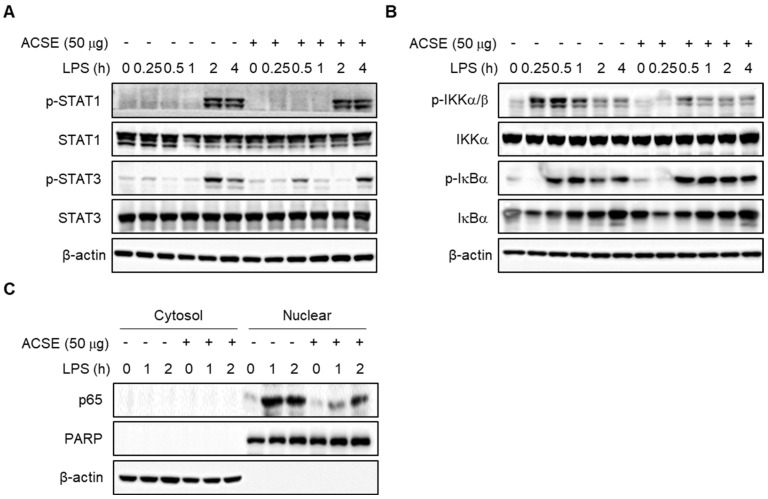
ACSE inhibits the NF-κB signaling pathway in LPS-stimulated macrophages. J774 cells were pretreated with 50 µg/mL of ACSE and then stimulated with 1 µg/mL of LPS for the indicated hours. (**A**,**B**) The effects of ACSE on the phosphorylation of STAT1/STAT3 (**A**) and IKKα/β and IκBα (**B**). (**C**) The effect of ACSE on the nuclear translocation of NF-κB p65. PARP and β-actin were used as loading controls for the nuclear and cytosolic fractions, respectively.

Because the expression of several inflammatory cytokines is regulated by the transcription factor STAT1/3 and NF-κB [9,10], we next investigated the involvement of these transcription factors in the anti-inflammatory effects of ACSE. Pretreatment with ACSE did not affect the phosphorylation of STAT1 and STAT3 (Figure 5A). However, ACSE pretreatment significantly reduced the LPS-induced phosphorylation of IKKα/β in J774 macrophages in a time-dependent manner (Figure 5B). Furthermore, ACSE markedly suppressed the LPS-mediated translocation of p65 in J774 macrophages (Figure 5C). These results suggest that ACSE attenuates LPS-mediated inflammation responses by inhibiting NF-κB activation in macrophages.

### 2.5. LC-MS Base Ion Peak Chromatogram of Astragali Complanate Semen

**Figure 6:**
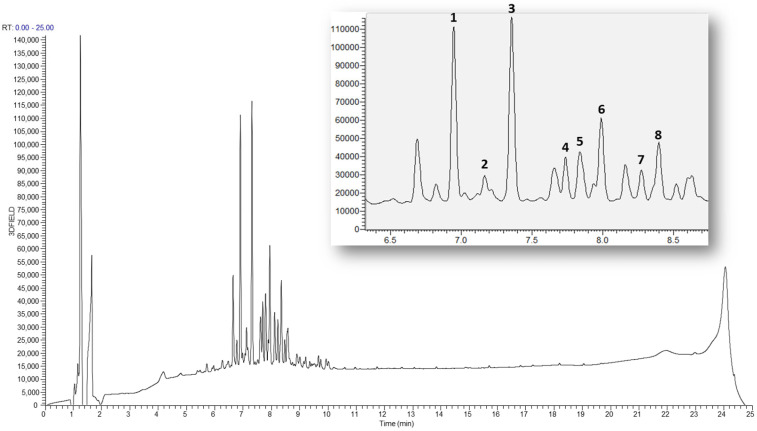
LC-MS profile of the 70% EtOH extract of Astragali Complanati Semen. Myricetin 3-*O*-β-d-xylopyranosyl(1→2)-β-d-glucopyranoside (**1**), myricetin 3-*O*-rutinoside (**2**), myricetin 3-*O*-β-d-glucopyranoside (**3**), 3′-Hydroxy-4′-methoxyisoflavone-7-*O*-β-d-glucopyranoside (**4**), laricitin 3-*O*-β-d-glucopyranoside (**5**), myricetin 3′-*O*-β-d-glucopyranoside (**6**), kaempferol 3-*O*-β-d-glucopyranoside (**7**), complanatuside (**8**). The panel on the right shows the enlarged LC-MS data of the peaks of compounds **1**–**8**.

The 70% ethanol extract of Astragali Complanate Semen was analyzed via HPLC-mass spectrometry in the positive ion mode and eight main peaks were identified by comparing the retention times with the reference compounds isolated from the title plant (Figure 6).

## 3. Discussion

Sepsis is a systemic inflammatory syndrome that results in multiple-organ failure caused by a dysregulated host immune response to microbial infection. Sepsis is a major health problem and a leading cause of mortality and fatal diseases worldwide. Despite advances in medical treatment, effective drugs for sepsis are still lacking [2,3]. Research on sepsis treatment is being conducted using diverse strategies such as discovering new biomarkers [22,23], immune regulation, inflammation suppression [24,25], and the management of antibiotic resistance [26]. Natural products provide a promising strategy for treating sepsis owing to their multiple biological activities, including antimicrobial, anti-inflammatory, and immunomodulatory activities [2,27]. ACSE is an herbal medicine used to treat muscle, renal, liver, and reproductive system diseases [15]. ACSE has several pharmacological activities, including antihypertensive, antifibrosis, antioxidant, antiaging, and anti-diabetic effects [17,18,19]. However, the effects of ACSE on sepsis have not been reported. Here, we newly discovered that ACSE has anti-inflammation and antiseptic effects. This study showed that ACSE significantly protects against sepsis by inhibiting the excessive production of inflammatory cytokines and mediators in mice with sepsis induced by CLP and LPS. Administration of ACSE reduced the mortality rate in septic mice. AST and ALT are transaminases that reflect liver damage and are the primary indicators of liver function in clinical practice [28,29]. We explored the effects of ACSE on sepsis-induced liver damage using these indicators. The results show that ACSE remarkably reduced serum levels of AST and ALT in mice with CLP- and LPS-induced sepsis, suggesting that ACSE protects against liver damage caused by CLP- and endotoxin-induced septic shock.

Macrophages play an important role in various pathophysiological processes during sepsis [30]. When infected with pathogens, macrophages mediate inflammatory responses, including the production of cytokines and chemokines. During sepsis, macrophages produce excessive amounts of inflammatory factors that cause systemic inflammation [11]. Excessive production of proinflammatory cytokines is significantly associated with the prognosis of patients [5]. Therefore, inhibition of inflammatory cytokines may be a promising strategy for treating sepsis. In the present study, because ACSE increased survival rates and alleviated liver injury in CLP- and LPS-induced septic mice, we expected that ACSE would suppress the expression of inflammatory cytokines in sepsis. Therefore, we validated the anti-inflammatory efficacy of ACSE in vitro and in vivo. As expected, ACSE inhibited the LPS-induced expression of IL-1β, IL-6, and TNF-α in macrophages. Furthermore, ACSE alleviated serum levels of IL-1β, IL-6, TNF-α, and NO in CLP- and LPS-induced septic mice. Therefore, we speculate that ACSE exerts protective effects against septic shock by suppressing the excessive inflammatory response of macrophages.

The NF-κB signaling is one of the central pathways of the pathogenesis of sepsis [31,32]. NF-κB is an inducible transcription factor that regulates the expression of several genes involved in inflammatory responses [33]. NF-κB is activated by the inducible proteolytic degradation of IκB proteins through phosphorylation by the IκB kinase (IKK) complex, which is activated by various microbial pathogens. Upon activation, IKK phosphorylates IκBα and promotes its degradation, resulting in translocation to the nucleus of NF-κB members, which induces the expression of inflammatory-related genes [34]. IKK inhibitors attenuate sepsis-induced excessive inflammation and cardiac dysfunction [35,36]. Because ACSE inhibited the expression of LPS-induced inflammatory mediators in macrophages, we evaluated the effect of ACSE on LPS-induced NF-κB activation. As expected, our data indicated that ACSE attenuated the LPS-mediated phosphorylation of IKK and the nuclear translocation of the p65 NF-κB subunit. Consequently, ACSE suppressed the expression of NF-κB downstream target genes, including iNOS, COX-2, IL-1β, IL-6, and TNF-α in LPS-stimulated macrophages. These findings imply that the NF-κB signaling pathway may participate in the antiseptic effect of ACSE.

## 4. Materials and Methods

### 4.1. Preparation of ACSEs

The 70% ethanol extract of ACS (KOC-70E-380), a lyophilized powder, was obtained from KOC Biotech Co. (Daejeon, Republic of Korea). The ACS powder was suspended in a 10% DMSO, filtered using a 0.22 μm syringe filter, and then stored at −80 °C.

### 4.2. Cell Culture

J774A.1 cells (murine macrophage cell line) were purchased from the American Type Culture Collection (Rockville, MD, USA) and cultured in DMEM (Gibco, Grand Island, NY, USA) supplemented with 10% fetal bovine serum and 1% penicillin/streptomycin (all from Gibco, Grand Island, NY, USA) at 37 °C in a humidified incubator with 5% CO_2_.

### 4.3. Animal Studies

All animal work was approved by the Institutional Animal Care and Use Committee of the Korea Research Institute of Bioscience and Biotechnology (KRIBB; Deajeon, Republic of Korea) and was performed according to the institutional guidelines at KRIBB (KRIBB-AEC-21041). Specific pathogen-free male C57BL/6 mice, 7–8 weeks of age, were obtained from KRIBB (Cheongju, Republic of Korea). They were housed in a humidity- and temperature-controlled room (22 ± 2 °C) with a 12/12-h light/dark cycle at the Animal Experiment Center of KRIBB (Daejeon, Republic of Korea). For the CLP-induced septic shock mouse model, mice were randomly separated into four groups (sham, CLP/PBS, CLP/ACSE-20, and CLP/ACSE-50; n = 10 per group). The mice were fasted overnight and then administrated PBS, 20 mg/kg ACSE, or 50 mg/kg ACSE through oral gavage for 7 days. On the next day, CLP was performed. Briefly, mice were anesthetized using avertin (500 mg/kg; Sigma-Aldrich, St. Louis, MO, USA). The cecum was exteriorized after creating a 1 cm incision in the peritoneum. Around 1 cm of the distal cecum was ligated with a silk suture, and then this ligated portion of the cecum was pierced with a 23-gauge needle in a single pass. The ligated and punctured cecum was then placed back into the abdomen, and the peritoneum was sewn with a silk suture and then disinfected with povidone. The cecum was exteriorized for mice in the sham group without ligation and puncture. The cecum was returned to the abdomen, and the peritoneum was sewn with a silk suture and then disinfected with povidone. For the LPS-induced septic shock model, mice were randomly separated into four groups (control, LPS/PBS, LPS/ACSE-20, and LPS/ACSE-50; n = 10 per group). The mice were fasted overnight and then administrated PBS, 20 mg/kg ACSE, or 50 mg/kg ACSE through oral gavage for 7 days. On the next day, mice were injected with LPS (10 mg/kg). Mice were monitored for survival for 7 days after LPS treatment or CLP operation. The concentration of LPS injected into mice (10 mg/kg) was determined from previous studies [37,38,39].

### 4.4. Blood Sample Collection

For blood sample collection, 18 h after the LPS treatment or CLP operation were ad-ministered, blood samples were obtained from mice through cardiac blood sampling after anesthesia with avertin. Collected blood samples were centrifuged at 2500× *g* for 25 min to separate the serum and then stored at −80 °C until the analysis of aspartate aminotransferase (AST), alanine aminotransferase (ALT), creatine kinase (CK), and cytokines levels. Levels of AST, ALT, and CK were determined using an automated blood chemistry analyzer (AU480; Beckman Coulter, Krefeld, Germany).

### 4.5. Cell Viability

J774.A.1 cells were seeded into a 96-well plate at a density of 1 × 10^4^ cells/well and grown for 24 h, and then cells were treated with ACSE for 24 h. Cell viability was measured using a WST-1 assay (Roche, Pleasanton, CA, USA) according to the manufacturer’s protocol. Cell absorbance was measured at 450 nm using a microplate reader.

### 4.6. NO Analytical Measurements

The concentration of NO was quantified by 100 μL of either the culture supernatants or serum samples from septic mice induced by LPS and CLP as previously described [37]. Briefly, these samples were mixed with the same amount of Griess reagent (0.1% *N*-(1-naphthyl) ethylenediamine dihydrochloride and 1% sulfanilamide in 5% phosphoric acid) and allowed to react for 10 min. In the case of serum samples, measurements were conducted following dilution with the appropriate ratio of sample-dilution buffer. Subsequently, absorbance was measured at 562 nm using a microplate reader. The NO concentration in each sample was determined with reference to a sodium nitrite (NaNO_2_) standard curve.

### 4.7. Enzyme-Linked Immunosorbent Assay (ELISA)

The quantitation of cytokines was conducted using a mouse TNF-α (DY-410), IL-1β (DY-401), and IL-6 (DY-406) DuoSet ELISA kit. Supernatant samples were collected from the cell culture supernatants and serum samples of septic mice induced by LPS and CLP. All ELISA kits were purchased from R&D Systems (Minneapolis, MN, USA) and used according to the manufacturer’s instructions.

### 4.8. Immunoblotting

Cells were solubilized in RIPA lysis buffer. The extracted proteins were quantified using a BCA assay (Intron biotechnology, Gyeonggi, Republic of Korea), and protein lysates were fractionated using 6–15% SDS-PAGE. The proteins on the SDS-PAGE gels were transferred to PVDF membranes. Then, membranes blocked with 5% skim milk were incubated with the following primary antibodies: iNOS (SC-650), β-actin (SC-47778), Cox2 (#12282), phospho-IKKα/β (#2697), IKKα (#61294), phospho-IκBα (#2859), IκBα (#9242), phospho-STAT1 (#9167), STAT1 (#9172), phospho-STAT3 (#9131), STAT3 (#9132), phospho-p65 (#3033), and PARP (#9532). After washing, the membranes were incubated with HRP-conjugated secondary antibodies and visualized with Chemi-doc equipment with enhanced chemiluminescent detection solution (Clarity Western ECL; Bio-Rad Laboratories, Inc., Hercules, CA, USA).

### 4.9. RT-PCR and Quantitative Real-Time PCR Analysis

Total RNA was prepared from cells using Trizol (Invitrogen, Carlsbad, CA, USA), and the concentration was quantified by a NanoVue Plus™ spectrophotometer (Biochrom, Holliston, MA, USA) as previously described [37]. Reverse transcription was conducted using a reverse transcription kit (Promega, Madison, WI, USA). PCR was performed using a 2X premix from Enzynomics (Daejeon, Republic of Korea). GAPDH was used as a normalization control. The PCR products were electrophoresed on a 1.5% agarose gel containing ethidium bromide and captured using photographic UV illumination. Real-time RT PCR was performed in triplicate using an AccuPower GreenStar qPCR Master Mix (Bioneer, Daedeok-gu, Daejeon, Republic of Korea). Gene expression levels were determined according to the 2^−ΔCt^ method, and samples were normalized with the housekeeping gene, GAPDH. All primers were synthesized by Bioneer, and the sequences of the primers are described in Table S1.

### 4.10. LC-MS Analysis

A liquid chromatography–mass spectrometer (LC-MS) was operated with an LTQXL linear ion trap (Thermo Scientific, Rockford, IL, USA) equipped with an electro-spray ionization (ESI) source that was coupled to a rapid separation LC (RSLC; Ultimate 3000, Thermo Scientific) system (ESI-LC-MS).

### 4.11. Statistical Analysis

Quantitative data were presented as mean ± standard deviation and analyzed using a two-tailed unpaired Student’s *t*-test to assess the differences between groups. Statistical significance was considered when the *p*-value was smaller than 0.05, indicating significant differences between the groups.

## 5. Conclusions

In conclusion, we elucidated the antiseptic efficacy of ACSE and its underlying mechanism for the first time. This study indicates that ACSE attenuates the production of inflammatory mediators by suppressing the NF-κB pathway, resulting in the alleviation of septic shock. Although further experiments are necessary to explore other signaling pathways regulated by ACSE, our findings suggest that ACSE is a promising candidate for treating sepsis.

## Data Availability

The original contributions presented in the study are included in the article/Supplementary Materials; further inquiries can be directed to the corresponding authors.

## Supplementary Materials

The supporting information can be downloaded at: https://www.mdpi.com/article/10.3390/ijms25010384/s1.

## Abbreviations

ACS	Astragali Complanati Semen
ACSE	Astragali Complanati Semen extract
ALT	Alanine aminotransferase
AST	Aspartate aminotransferase
CLP	Cecal ligation and puncture
COX-2	Cyclooxygenase-2
CRP	C-reactive protein
DAMP	Damage-associated molecular pattern
IKK	IκB kinase
IL-1β	Interleukin-1β
IL-6	Interleukin-6
iNOS	Inducible nitric oxide synthase
LC-MS	Liquid chromatography-mass spectrometry
LPS	Lipopolysaccharide
MAPK	Mitogen-activated protein kinase
MODS	Multiple organ dysfunction syndrome
NF-κB	Nuclear factor-κB
NO	Nitric oxide
PAMP	Pathogen-associated molecular pattern
PRR	Pattern recognition receptor
STAT	Signal transducer and activator of transcription
TLR4	Toll-like receptor 4
TNF-α	Tumor necrosis factor-α
