# Supplementary material for: Astragalus Complanatus Ethanol Attenuates Septic Shock by Exerting Anti-Inflammatory Effects on Macrophages
Source: Int J Mol Sci. 2023 Dec 27;25(1):384. doi: 10.3390/ijms25010384 (PMC10778658; doi:10.3390/ijms25010384)
Supplement: Supplementary file 1 [file ijms-25-00384-s001.zip › ijms-2768058-supplementary.pdf]

Table S1. Primer list for RT-PCR.

Gene Name	(5') Forward Primers (3')	(5') Reverse Primers (3')
**iNOS**	CAAGCACCTTGGAAGAGGAG	AAGGCCAAACACAGCATACC
**COX2**	GCTGTACAAGCAGTGGCAAA	TTCTGCAGCCATTTCCTTCT
**TNF-α**	ACGGCATGGATCTCAAAGAC	TGAGATAGCAAATCGGCTGAC
**IL-1β**	GAGTGTGGATCCCAAGCAAT	CTTGTGCTCTGCTTGTGAGG
**IL-6**	CTGATGCTGGTGACAACCAC	TCCACGATTTCCCAGAGAAC
**GAPDH**	ACCCAGAAGACTGTGGATGG	ACACATTG GGGGTAGGAACA
